# Reduced complications and mortality after admission for head and neck cancer in patients with previous dental scaling: a retrospective cohort study based on real-world data

**DOI:** 10.1371/journal.pone.0332992

**Published:** 2025-10-03

**Authors:** Che-Hsuan Lin, Chuen-Chau Chang, Hsun-Hua Lee, Yi-Sheng Chou, Chia-Yen Lee, Chun-Chieh Yeh, Ta-Liang Chen, Chien-Chang Liao

**Affiliations:** 1 Department of Otolaryngology, Taipei Medical University Hospital, Taipei, Taiwan; 2 Department of Otolaryngology, School of Medicine, College of Medicine, Taipei Medical University, Taipei, Taiwan; 3 Department of Anesthesiology, Taipei Medical University Hospital, Taipei, Taiwan; 4 Health Policy Research Center, Taipei Medical University Hospital, Taipei, Taiwan; 5 Department of Anesthesiology, School of Medicine, College of Medicine, Taipei Medical University, Taipei, Taiwan; 6 Department of Neurology, Taipei Medical University Hospital, Taipei, Taiwan; 7 Department of Neurology, School of Medicine, College of Medicine, Taipei Medical University, Taipei, Taiwan; 8 Department of Hematology and Oncology, Taipei City Hospital, Renai Branch, Taipei, Taiwan; 9 Department of Dentistry, Taiwan Adventist Hospital, Taipei, Taiwan; 10 Department of Surgery, China Medical University Hospital, Taichung, Taiwan; 11 Department of Surgery, University of Illinois, Chicago, Illinois, United States of America; 12 Department of Anesthesiology, Wang Fang Hospital, Taipei Medical University, Taipei, Taiwan; 13 Research Center of Big Data and Meta‑Analysis, Wan Fang Hospital, Taipei Medical University, Taipei, Taiwan; 14 School of Chinese Medicine, College of Chinese Medicine, China Medical University, Taichung, Taiwan; University of California, Davis, UNITED STATES OF AMERICA

## Abstract

**Background:**

The association between oral health and cancer outcomes remains unclear. The purpose of this study was to evaluate the complications and mortality after admission of head and neck cancer (HNC) in patients with and without dental scaling (DS).

**Methods:**

We used data from public health insurance and identified 121,973 patients with admission of HNC aged ≥ 18 years who received inpatient care in 2006–2020. The outcomes during the admission of HNC were compared between patients who had received DS or not within the previous 24 months before admission. The adjusted odds ratios (HRs) and 95% confidence intervals (CIs) of complications and mortality associated with DS were analyzed in the multivariate Cox proportional regression models.

**Results:**

We found that DS was significantly associated with reduced risks of septicemia (OR 0.84, 95% CI 0.81–0.88), stroke (OR 0.87, 95% CI 0.80–0.95), pneumonia (OR 0.88, 95% CI 0.84–0.91), urinary tract infection (OR 0.88, 95% CI 0.80–0.97), and 30-day in-hospital mortality (OR 0.88, 95% CI 0.85–0.92). Compared with HNC patients without DS, HNC patients with DS had a shortened length of hospital stay (p < 0.0001), decreased medical expenditures (p < 0.0001), and reduced risks of intensive care (OR 0.92, 95% CI 0.89–0.95) after admission of HNC.

**Conclusion:**

In conclusion, we suggested that HNC patients who received DS had reduced complications and mortality compared with those without DS. It is essential to interpret this association with caution due to the confounding factors involved. Our study implied the possibility that clinical physicians may encourage HNC patients to receive regular DS.

## Introduction

Head and neck cancer (HNC) is one of the most common cancers in the world, with 1.1 million new diagnoses reported annually [1–5]. The incidence of HNC in the United States increased between 1990 and 2017 [3]. The mortality burden of head and neck cancer is increasing and disproportionately affects those in low- and middle-income countries and regions with limited surgical workforces [5]. It is considered a major public health problem due to its high prevalence and mortality. Several common risk factors for HNC were identified, including poor oral hygiene, smoking, betel nut chewing, alcohol consumption, and infections of Epstein‒Barr virus and human papillomavirus [4,6]. A multidisciplinary approach was required for HNC treatment planning, including surgeons, radiation oncologists, medical oncologists, nutritionists, speech/swallowing pathologists and dentists. According to the National Comprehensive Cancer Network guidelines [7], the treatment options will vary based on different sites and stages, including surgery, radiotherapy and systemic therapy.

Poor oral hygiene is considered an important risk factor for HNC [1]. Dental calculus can lead to overgrowth of pathogenic bacteria in the oral cavity and irritation of the oral mucosa and further induce inflammation [8]. In addition, poor oral hygiene may also promote the progression of HNC [9]. It promotes the entry of oral microbes into the bloodstream, which in turn triggers a systemic inflammatory response [8,9]. Dental scaling (DS) can reduce systemic inflammation, further prevent loss of kidney function [10], and decrease the risk of cardiovascular disease [11] and infective endocarditis [12].

It is known that adequate oral hygiene plays an important protective role in the risk of HNC [13]. Dental management before radiation therapy can effectively reduce the occurrence of various complications after radiation therapy [14]. However, the benefits of DS in patients with HNC during the treatment period are misunderstood. The role of DS in improving treatment complications such as infections, cardiovascular diseases and survival rates in patients with HNC has not been discussed in depth. In this study, we used population-based insurance data to evaluate the beneficial effects of regular DS on the outcomes of hospitalized patients with HNC.

## Methods

### Ethics statement

With reference to the Declaration of Helsinki, patient identification was decoded and scrambled in the insurance research database for the protection of the patient’s right to privacy. According to the regulation of the Ministry of Health and Welfare in Taiwan, informed consent from the study participants in this study was waived by the ethics committee because of the use of decoded and scrambled patient identification and. The results of analysis was reviewed and assessed by the Ministry of Health and Welfare. Our study was also reviewed and evaluated by the Institutional Review Board of Taipei Medical University (TMU-JIRB-202203134; TMU-JIRB-202006057).

### Study design

This insurance data were accessed for research purposes in 04/04/2023. To approach a large representative sample, we used research data from the National Health Insurance (details of this database were described in previous studies) to identify the cohort with HNC [10–12,15,16]. Patients aged more than 18 years who first underwent inpatient care due to HNC were screened and selected as eligible subjects in this study. Patients with HNC who had a previous history of other cancers were excluded from this study. Overall, we identified 121,973 patients with HNC admission in 2006–2020, and 45,849 of them had received the medical service of DS provided by dentists in the clinical settings during the 24 months before hospitalization. The outcomes of this study were complications and mortality during the hospitalization of HNC that were compared between patients with and without DS.

### Criteria and definition

In the insurance research database, we collected information and data on gender (male and female), age (18−29, 30−39, 40−49, 50−59, 60−69, 70−79, and ≥80 years), state of income (low income or not low income), medical conditions (including hypertension, mental disorders, diabetes, ischemic heart disease, hyperlipidemia, chronic obstructive pulmonary disease, liver cirrhosis, heart failure, and renal dialysis), volume of hospital (low, medium, and high), and medical use (emergency care and hospitalization). To strictly identify the medical conditions of patients with HNC, we used previous definitions to evaluate medical care with the physician’s primary diagnosis before HNC hospitalization within 24 months. ICD-9-CM and ICD-10-CM were used to identify medical conditions and types of HNC. The use of emergency care and inpatient care were also considered important baseline characteristics that may affect the outcomes after HNC hospitalization. The types of HNC included oral cancer, oropharyngeal cancer, nasopharyngeal cancer, hypopharyngeal cancer, other cancers in the lip, oral cavity and pharynx, nasal and sinus cancer, and laryngeal cancer.

### Statistical analysis

By using chi-square tests and t tests, we analyzed and compared the baseline characteristics (including continuous variables and categorical variables) of HNC patients with and without DS. To assess the potential effects of DS on complications and mortality after HNC hospitalization, we calculated adjusted odds ratios (ORs) and 95% confidence intervals (CIs) using multiple logistic regressions and adjusted for potential confounding factors listed in the baseline characteristics. We further applied stratified analysis to evaluate the association between DS and adverse events after HNC hospitalization in age groups, sexes, income status, volume of hospital, previous hospitalizations, previous emergency visits, and history of diseases. We additionally assessed the effects of visiting frequency and time period of receiving DS on adverse events after HNC hospitalization by calculating ORs and 95% CIs in the multiple logistic regressions.

## Results

The usage of DS within the previous 24 months among 121,973 HNC patients was 37.6% (Table 1), and patients with DS had higher proportions of female (p < 0.0001), younger age (p < 0.0001), stay in high volume of hospital (p < 0.0001), nasopharyngeal cancer (p < 0.0001), hypertension (p < 0.0001), mental disorders (p < 0.0001), ischemic heart disease (p = 0.0002), and hyperlipidemia (p < 0.0001) than those without use of DS. Compared with patients who did not use DSs, patients who used DSs had lower proportions of baseline characteristics, such as low income (<0.0001), hospitalizations ≥3 visits (<0.0001), emergency care ≥3 visits (<0.0001), chronic obstructive pulmonary disease (<0.0001), liver cirrhosis (p = 0.0221), and heart failure (p = 0.0015).

**Table 1:** Characteristics of patients with head and neck cancer who received dental scaling or not.

	No DS (N = 76124)		DS (N = 45849)		p-value
Sex	n	(%)	n	(%)	<0.0001
Female	10120	(13.3)	7741	(16.9)	
Male	66004	(86.7)	38108	(83.1)	
Age, years					<0.0001
18-29	660	(0.9)	621	(1.4)	
30-39	4328	(5.7)	3325	(7.3)	
40-49	15254	(20.0)	9916	(21.6)	
50-59	24314	(31.9)	14741	(32.2)	
60-69	18571	(24.4)	10851	(23.7)	
70-79	9136	(12.0)	4745	(10.4)	
≥80	3861	(5.1)	1650	(3.6)	
Low income					<0.0001
No	70981	(93.2)	43353	(94.6)	
Yes	5143	(6.8)	2496	(5.4)	
Volume of hospital					<0.0001
Low	6027	(7.9)	3306	(7.2)	
Medium	25185	(33.1)	13892	(30.3)	
High	44912	(59.0)	28651	(62.5)	
Number of hospitalizations					<0.0001
0	31161	(40.9)	20010	(43.6)	
1	15674	(20.6)	9466	(20.7)	
2	8269	(10.9)	4729	(10.3)	
≥3	21020	(27.6)	11644	(25.4)	
Number of emergency visits					<0.0001
0	42039	(55.2)	26994	(58.9)	
1	14143	(18.6)	8243	(18.0)	
2	7275	(9.6)	3977	(8.7)	
≥3	12667	(16.6)	6635	(14.5)	
Types of cancer					<0.0001
Oral cancer	43709	(57.4)	25989	(56.7)	
Oropharyngeal cancer	5737	(7.5)	3176	(6.9)	
Nasopharyngeal cancer	10886	(14.3)	8501	(18.5)	
Hypopharyngeal cancer	7927	(10.4)	3534	(7.7)	
Other ENT cancer	610	(0.8)	389	(0.9)	
Nasal and sinus cancer	1667	(2.2)	1157	(2.5)	
Laryngeal cancer	5588	(7.3)	3103	(6.8)	
Medical conditions					
Hypertension	15965	(21.0)	10173	(22.2)	<0.0001
Mental disorders	14122	(18.6)	9169	(20.0)	<0.0001
Diabetes	11128	(14.6)	6698	(14.6)	0.9640
Ischemic heart disease	4640	(6.1)	3037	(6.6)	0.0002
Hyperlipidemia	3373	(4.4)	2584	(5.6)	<0.0001
COPD	5000	(6.6)	2607	(5.7)	<0.0001
Liver cirrhosis	3345	(4.4)	1889	(4.1)	0.0221
Heart failure	985	(1.3)	499	(1.1)	0.0015
Renal dialysis	656	(0.9)	328	(0.7)	0.0056

COPD, chronic obstructive pulmonary disease; DS, dental scaling.

After adjustment for the potential confounding factors listed in Table 1, patients who received DS had decreased risks of pneumonia (OR 0.88, 95% CI 0.84–0.91), septicemia (OR 0.84, 95% CI 0.81–0.88), urinary tract infection (OR 0.88, 95% CI 0.80–0.97), stroke (OR 0.87, 95% CI 0.80–0.95), and 30-day mortality (OR 0.88, 95% CI 0.85–0.92) compared with those without DS (Table 2). Decreased use of intensive care (OR 0.92, 95% CI 0.89–0.95), length of hospital stay (p < 0.0001), and medical expenditure (p < 0.0001) were also found in patients who used DS compared with patients without DS.

**Table 2:** Adverse outcomes after admission of head neck cancer in patients with and without dental scaling.

	No DS (N = 76124)		DS (N = 45849)		Risk of outcomes	
	Events	%	Event	%	OR	(95% CI)*
30-day in-hospital mortality	8905	11.7	4391	9.6	0.88	(0.85-0.92)
Complications						
Pneumonia	9242	12.1	4431	9.7	0.88	(0.84-0.91)
Septicemia	6149	8.1	2935	6.4	0.84	(0.81-0.88)
Stroke	1595	2.1	769	1.7	0.87	(0.80-0.95)
Urinary tract infection	1386	1.8	675	1.5	0.88	(0.80-0.97)
Acute myocardial infarction	126	0.2	52	0.1	0.74	(0.54-1.03)
Stay in intensive care unit	14305	18.8	7810	17.0	0.92	(0.89-0.95)
Medical expenditure, USD^†^	4341 ± 4834		4073 ± 4648		p < 0.0001	
Length of hospital stay, days^†^	12.7 ± 12.0		11.6 ± 11.0		p < 0.0001	

CI, confidence interval; DS, dental scaling; OR, odds ratio.

* Adjusted for all covariates listed in Table 1.

† Mean±SD; regular dental scaling was associated with medical expenditure (beta = −751.7, p < 0.0001) and length of hospital stays (beta = −2.27, p < 0.0001) after adjusted all covariates listed in Table 1 in the multiple linear regressions.

In Table 3, the decreased risk of post-HNC adverse events (included) was associated with DS in females (OR 0.85, 95% CI 0.78–0.93), males (OR 0.85, 95% CI 0.82–0.87), and patients aged 18−39 years (OR 0.84, 95% CI 0.74–0.96), 40−49 years (OR 0.86, 95% CI 0.80–0.92), 50−59 years (OR 0.84, 95% CI 0.80–0.89), 60−69 years (OR 0.83, 95% CI 0.77–0.88), and ≥70 years (OR 0.86, 95% CI 0.80–0.92). DS was negatively associated with post-HNC adverse events in patients with various characteristics, such as low income (OR 0.85, 95% CI 0.75–0.95), no low income (OR 0.84, 95% CI 0.82–0.87), stay in low (OR 0.83, 95% CI 0.75–0.92), medium (OR 0.82, 95% CI 0.78–0.86), and high volume of hospital (OR 0.87, 95% CI 0.83–0.90).

**Table 3:** The stratified analysis for the association between dental scaling and adverse events after head and neck cancer.

		Adverse events^*^			
		n	Events	Rate, %	OR	(95% CI)†
Female	No DS	10120	2120	21.0	1.00	(reference)
	DS	7741	1282	16.6	0.85	(0.78-0.93)
Male	No DS	66004	18026	27.3	1.00	(reference)
	DS	38108	8656	22.7	0.85	(0.82-0.87)
Age 18–39 years	No DS	4988	921	18.5	1.00	(reference)
	DS	3946	590	15.0	0.84	(0.74-0.96)
Age 40–49 years	No DS	15254	3883	25.5	1.00	(reference)
	DS	9916	2134	21.5	0.86	(0.80-0.92)
Age 50–59 years	No DS	24314	6245	25.7	1.00	(reference)
	DS	14741	3095	21.0	0.84	(0.80-0.89)
Age 60–69 years	No DS	18571	4789	25.8	1.00	(reference)
	DS	10851	2298	21.2	0.83	(0.77-0.88)
Age ≥ 70 years	No DS	12997	4308	33.2	1.00	(reference)
	DS	6395	1821	28.5	0.86	(0.80-0.92)
No low income	No DS	70981	18449	26.0	1.00	(reference)
	DS	43353	9244	21.3	0.84	(0.82-0.87)
Low income	No DS	5143	1697	33.0	1.00	(reference)
	DS	2496	694	27.8	0.85	(0.75-0.95)
Low volume of hospital	No DS	6027	2797	46.4	1.00	(reference)
	DS	3306	1274	38.5	0.83	(0.75-0.92)
Medium volume of hospital	No DS	25185	7578	30.1	1.00	(reference)
	DS	13892	3448	24.8	0.82	(0.78-0.86)
High volume of hospital	No DS	44912	9771	21.8	1.00	(reference)
	DS	28651	5216	18.2	0.87	(0.83-0.90)
Oral cancer	No DS	43709	10562	24.2	1.00	(reference)
	DS	25989	5072	19.5	0.83	(0.80-0.87)
Oropharyngeal cancer	No DS	5737	1856	32.4	1.00	(reference)
	DS	3176	820	25.8	0.79	(0.71-0.88)
Nasopharyngeal cancer	No DS	10886	2946	27.1	1.00	(reference)
	DS	8501	1982	23.3	0.89	(0.82-0.95)
Hypopharyngeal cancer	No DS	7927	3028	38.2	1.00	(reference)
	DS	3534	1264	35.8	0.91	(0.84-1.00)
Other ENT cancer	No DS	610	128	21.0	1.00	(reference)
	DS	389	53	13.6	0.78	(0.52-1.15)
Nasal and sinus cancer	No DS	1667	406	24.4	1.00	(reference)
	DS	1157	245	21.2	0.91	(0.74-1.12)
Laryngeal cancer	No DS	5588	1220	21.8	1.00	(reference)
	DS	3103	502	16.2	0.78	(0.69-0.89)

CI, confidence interval; DS, dental scaling; ENT, ear, nose, and throat; OR, odds ratio.

* Adverse events included with 30-day in-hospital mortality, pneumonia, septicemia, stroke, urinary tract infection, and acute myocardial infarction.

† Adjusted for all covariates listed in Table 1.

In Table 4, the adjusted ORs of post-HNC adverse events for patients with recent DS within 1, 3, and 6 months were 0.58 (95% CI 0.51–0.66), 0.64 (95% CI 0.59–0.69), and 0.71 (95% CI 0.67–0.75), respectively, compared with those who had no DS. We also found that there was a biological gradient relationship between the visit frequency of DS and reduced post-HNC adverse events (p for trend <0.0001). Regular use of DS (≥4 visits within 24 months) was significantly associated with reduced post-HNC adverse events (OR 0.61, 95% CI 0.53–0.72). Table 5 showed the reduced adverse events after admission of head and neck cancer associated with different time period of dental scaling. The results showed that dental scaling within 6 months prior to admission for head and neck cancer was associated with reduced adverse outcomes, compared with those who did not receive routine dental care within the previous 12 months (OR 0.69, 95% CI 0.65–0.73), 24 months (OR 0.71, 95% CI 0.67–0.75), 36 months (OR 0.71, 95% CI 0.67–0.75), 48 months (OR 0.71, 95% CI 0.67–0.76), and 60 months (OR 0.72, 95% CI 0.68–0.76).

**Table 4:** Reduced adverse events after admission of head and neck cancer associated with frequency and time period of dental scaling.

	Adverse events*				
	n	Events	Incidence, %	OR	(95% CI)^†^
No dental scaling	76124	20146	26.5	1.00	(reference)
Dental scaling in recent 1 month	2904	285	9.8	0.58	(0.51-0.66)
Dental scaling in recent 3 month	6256	815	13.0	0.64	(0.59-0.69)
Dental scaling in recent 6 month	10208	1674	16.4	0.71	(0.67-0.75)
Dental scaling = 1 visits	28116	6699	23.8	0.89	(0.86-0.92)
Dental scaling = 2 visits	11332	2236	19.7	0.80	(0.76-0.84)
Dental scaling = 3 visits	4760	783	16.5	0.74	(0.67-0.80)
Dental scaling ≥4 visits	1641	220	13.4	0.61	(0.53-0.72)

CI, confidence interval; DS, dental scaling; OR, odds ratio.

* Adverse events included with 30-day in-hospital mortality, pneumonia, septicemia, stroke, urinary tract infection, acute myocardial infarction.

† Adjusted for all covariates listed in Table 1.

**Table 5:** Reduced adverse events after admission of head and neck cancer associated with different time period of dental scaling.

	Adverse events*				
	n	Events	Incidence, %	OR	(95% CI)^†^
No DS in recent 12 month	78896	21350	27.1	1.00	(reference)
DS in recent 6 month	10208	1674	16.4	0.69	(0.65-0.73)
DS in recent 12 month	32122	6136	19.1	0.76	(0.74-0.79)
No dental scaling in recent 24 month	76124	20146	26.5	1.00	(reference)
DS in recent 6 month	10208	1674	16.4	0.71	(0.67-0.75)
DS in recent 12 month	32122	6136	19.1	0.78	(0.75-0.81)
DS in recent 24 month	45849	9938	21.7	0.84	(0.82-0.87)
No dental scaling in recent 36 month	75242	19815	26.3	1.00	(reference)
DS in recent 6 month	10208	1674	16.4	0.71	(0.67-0.75)
DS in recent 12 month	32122	6136	19.1	0.78	(0.75-0.81)
DS in recent 24 month	45849	9938	21.7	0.85	(0.82-0.87)
No dental scaling in recent 48 month	74356	19455	26.2	1.00	(reference)
DS in recent 6 month	10208	1674	16.4	0.71	(0.67-0.76)
DS in recent 12 month	32122	6136	19.1	0.79	(0.76-0.82)
DS in recent 24 month	45849	9938	21.7	0.85	(0.83-0.88)
No dental scaling in recent 60 month	73312	19036	26.0	1.00	(reference)
DS in recent 6 month	10208	1674	16.4	0.72	(0.68-0.76)
DS in recent 12 month	32122	6136	19.1	0.79	(0.76-0.82)
DS in recent 24 month	45849	9938	21.7	0.86	(0.83-0.88)

CI, confidence interval; DS, dental scaling; OR, odds ratio.

* Adverse events included with 30-day mortality, pneumonia, septicemia, stroke, urinary tract infection, and acute myocardial infarction.

† Adjusted for all covariates listed in Table 1.

## Discussion

In this retrospective cohort study consisting of 121,973 patients admitted for HNC, we investigated whether DS was significantly associated with decreased risks of complications and 30-day mortality even among those with coexisting medical conditions. Compared with patients without DS, patients with DS had a shortened length of hospital stay, decreased medical expenditures, and reduced risks of intensive care after admission related to HNC.

Previous studies have demonstrated that DS is associated with reduced stroke, urinary tract infection, pneumonia, sepsis, and all-cause mortality [10,16, 17–19]. DS treatment for periodontal disease was associated with a lower risk of further ischemic stroke events [16]. A recent study suggested that regular DS was associated with reduced complications and mortality in patients with stroke admission [20]. The findings of our study were consistent with the above reports showing that DS was associated with a 25% reduced risk of 30-day in-hospital mortality, a 32% reduced risk of septicemia, and a 31% reduced risk of stroke in patients with HNC.

A previous study suggested that poor oral hygiene is not only a risk factor but may also be a prognostic factor of HNC [9]. However, the effects of dental treatment on the survival and adverse events of patients with HNC have not been reported. In this study, the benefits of DS in patients with HNC during the treatment period were evaluated comprehensively. Our study may be the first to show the role of DS in improving complications such as pneumonia, septicemia, urinary tract infection, stroke, and survival rates in patients with HNC.

Regarding the association between DS and reduced post-HNC adverse events, we proposed some possible explanations as follows. First, people who receive regular DS may have better oral health knowledge, upper middle socioeconomic status, and good family support, which are also factors associated with outcomes of HNC [21–24]. Second, loss of teeth was significantly associated with oral cancer mortality, while regular DS was effective in preventing tooth loss [25]. Thus, we hypothesize that regular DS is beneficial in reducing post-HNC infections and mortality. Third, a previous study found a high systemic immune-inflammation index to be an independent factor for poor outcomes of HNC, including nutritional status, cancer stage, and overall survival [26]. A meta-analysis also suggested that systemic immune inflammation was considered a predictor of adverse outcomes in head and neck cancer prognosis [27]. Nonsurgical periodontal treatment, including DS, is effective in reducing serum leptin, interleukin 6, and levels of C-reactive protein [28]. It can also reduce oxidative stress in both smokers and nonsmokers [29]. Receiving DS may reduce systemic inflammation, gut dysbiosis, dissemination and subsequent infections in an immunosuppressive state [16,30–32]. Therefore, we considered it reasonable that regular DS can help to improve the inflammation status and further reduce adverse outcomes in patients with HNC.

In addition, poor oral hygiene may worsen survival in patients with HNC, and nutritional status in patients with HNC is related to survival and surgical outcome [9,33].

There was an obvious relationship between oral function and nutritional status [34]. Poor oral hygiene was associated with poorer nutritional status, while maintaining oral hygiene may contribute to better nutritional status and quality of life [35,36]. Therefore, we considered that regular DS is one method of maintaining oral hygiene that contributes to better nutritional status and may help reduce complications and mortality in patients with HNC.

To confirm the association between DS and post-NHC adverse events, we performed a sensitivity analysis to evaluate the effects of DS on post-NHC adverse events. The stratified analysis showed that DS was associated with a reduced risk of post-NHC adverse events in the various groups by age stratum, sex, and medical conditions. The different time periods (previous 1, 3, and 6 months) of DS before admission were associated with reduced post-NHC adverse events. The frequency of DS before admission was significantly associated with reduced post-NHC adverse events in this study. In addition, we performed propensity-score matching to balance the baseline characteristics, and the results also showed that patients with DS had lowered risks of adverse events after HNC admission compared with those who had no DS. From the viewpoint of epidemiology and statistics, we have more confidence to suggest that HNC patients who received DS had reduced adverse events compared with those who did not receive DS.

There were some limitations in the current study. First, we could not ensure that all people in the control group (without DS) had not received DS services because very few people who received DS services were not covered in the insurance program. This condition may lead to misclassification that causes the underestimation of the beneficial effects of DS on post-HNC complications and mortality. Second, we used insurance claims data that lacked details on sociodemographic information, lifestyle factors (such as smoking and alcohol consumption), and clinical examination data. Smoking and alcohol consumption are potential confounding factors in the association between dental scaling and post-HNC outcomes. We acknowledge that the lack of these data is a significant limitation that weakens the robustness of our findings. Third, people who received DS services may have better health knowledge, attitudes, and practices than those who had no DS. Our study was also limited to not adjusting for the above health-related factors in the final model. In addition, we could not exclude the possibility of overestimating the effects of DS on the outcomes of HNC in this study. To avoid overstatement or misrepresentation of findings, we conducted an additional analysis to compare the outcomes after HNC using various definitions of dental scaling and non-dental scaling groups. Finally, although we used multiple regression models to control potential confounders in this large-scale insurance database, residual confounding could not be completely excluded

In conclusion, we suggested that receiving DS regularly was significantly associated with reduced complications and mortality in patients admitted for HNC, even in those with coexisting medical conditions. It is essential to interpret this association with caution due to the confounding factors involved. The findings of our study provide references for health authorities and clinical physicians to encourage patients with HNC to receive regular DS against subsequent adverse events. Further clinical trials are encouraged to provide solid evidence to support our suggestions.

## Abbreviations

CI: Confidence interval
DS: Dental scaling
HNC: Head and neck cancer
OR: Odds ratio
